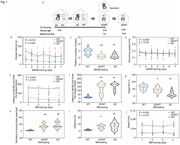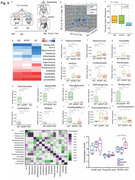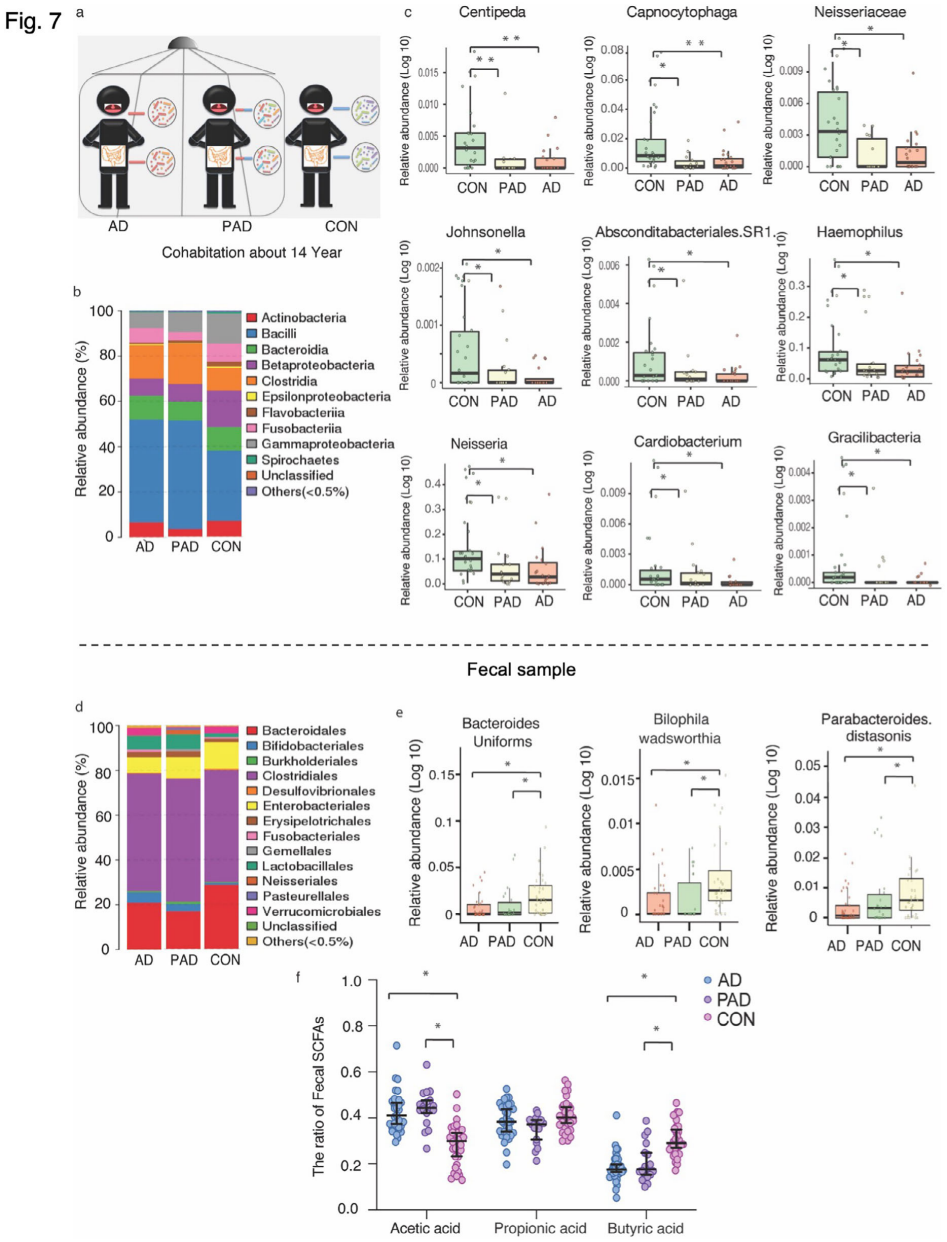# Transmission of Alzheimer’s disease‐associated microbiota dysbiosis and its impact on cognitive function: evidence from mice and patients

**DOI:** 10.1002/alz.093031

**Published:** 2025-01-03

**Authors:** Yiying Zhang, Yuan Shen, Wei Li, zhongcong xie

**Affiliations:** ^1^ Massachusetts General Hospital/Harvard Medical School, Charlestown, MA USA; ^2^ Anesthesia, Critical Care & Pain Medicine, Boston, MA USA

## Abstract

**Background:**

Spouses of Alzheimer’s disease (AD) patients are at a higher risk of developing incidental dementia. However, the causes and underlying mechanism of this clinical observation remain largely unknown. One possible explanation is linked to microbiota dysbiosis, a condition that has been associated with AD. However, it remains unclear whether gut microbiota dysbiosis can be transmitted from AD individuals to non‐AD individuals and contribute to the development of AD pathogenesis and cognitive impairment.

**Method:**

We, therefore, set out to perform both animal studies and clinical investigation by co‐housing wild‐type mice and AD transgenic mice, analyzing microbiota via 16S rRNA gene sequencing, measuring short‐chain fatty acid amounts, and employing behavioral test, mass spectrometry, site‐mutations and other methods.

**Result:**

The present study revealed that co‐housing between wildtype mice and AD transgenic mice or administrating feces of AD transgenic mice to wild‐type mice resulted in AD‐associated gut microbiota dysbiosis, Tau phosphorylation, and cognitive impairment in the wild‐type mice. Gavage with Lactobacillus and Bifidobacterium restored these changes in the wild‐type mice. The oral and gut microbiota of AD patient partners resembled that of AD patients but differed from healthy controls, indicating the transmission of microbiota. The underlying mechanism of these findings includes that the butyric acid‐mediated acetylation of GSK3β at lysine 15 regulated its phosphorylation at serine 9, consequently impacting Tau phosphorylation.

**Conclusion:**

This initial proof‐of‐concept study demonstrated the potential transmission of microbiota from AD patients or ADTg mice to non‐AD controls or WT mice housed together. This transmission may contribute to the development of AD‐associated microbiota dysbiosis and metabolite alterations, ultimately leading to AD pathogenesis and cognitive impairment. However, these findings should be validated in future studies. The results of this study are expected to drive further research to explore gut microbiota dysbiosis and its connection to AD in both preclinical and clinical contexts.